# Transmissive terahertz metasurfaces with vanadium dioxide split-rings and grids for switchable asymmetric polarization manipulation

**DOI:** 10.1038/s41598-022-07265-6

**Published:** 2022-03-03

**Authors:** Andriy E. Serebryannikov, Akhlesh Lakhtakia, Guy A. E. Vandenbosch, Ekmel Ozbay

**Affiliations:** 1 Division of Physics of Nanostructures, ISQI, Faculty of Physics, Adam Mickiewicz University, 61-614 Poznan, Poland; 2 Department of Engineering Science and Mechanics, The Pennsylvania State University, University Park, Pennsylvania, 16802 USA; 3 WaveCoRe research group, Electrical Engineering Department (ESAT), Katholieke Universiteit Leuven, 3001 Leuven, Belgium; 4 Nanotechnology Research Center (NANOTAM), National Institute of Materials Science and Nanotechnology (UNAM), Department of Physics, Department of Electrical Engineering, Bilkent University, 06800 Ankara, Turkey

**Keywords:** Optics and photonics, Metamaterials

## Abstract

Metasurfaces containing arrays of thermally tunable metal-free (double-)split-ring meta-atoms and metal-free grids made of vanadium dioxide (VO$_2$), a phase-change material can deliver switching between (1) polarization manipulation in transmission mode as well as related asymmetric transmission and (2) other functionalities in the terahertz regime, especially when operation in the transmission mode is needed to be conserved for both phases of VO$_2$. As the meta-atom arrays function as arrays of metallic subwavelength resonators for the metallic phase of VO$_2$, but as transmissive phase screens for the insulator phase of VO$_2$, numerical simulations of double- and triple-array metasurfaces strongly indicate extreme scenarios of functionality switching also when the resulting structure comprises only VO$_2$ meta-atoms and VO$_2$ grids. More switching scenarios are achievable when only one meta-atom array or one grid is made of VO$_2$ components. They are enabled by the efficient coupling of the geometrically identical resonator arrays/grids that are made of the materials that strongly differ in terms of conductivity, i.e. Cu and VO$_2$ in the metallic phase.

## Introduction

Metasurface research has been flourishing during the past decade, as quasiplanar designs enabling manipulation of the polarization state and wavefront are being investigated^1^. In particular, tunable metasurfaces have attracted a lot of attention in recent years. Various materials and tunability mechanisms have been proposed for use in metasurfaces designed to operate as spectral filters, polarization convertors, wave-front manipulating devices, etc.^4,5^. Transition from an insulator (I) phase to a metallic (M) phase, or vice versa, under the influence of a control parameter is a general feature of many natural materials^2,3^ that allows control of the permittivity tensor in the complex plane at a fixed frequency. It has particular significance for metasurfaces with switchable functionality.

Vanadium dioxide (VO$_2$) is an attractive phase-change material displaying a hysteretic insulator–metal–insulator transition^6–8^. The underlying monoclinic-to-tetragonal phase change occurs as the temperature *T* is raised from a value slightly lower than $58~^\circ \mathrm{C}$ to a value slightly above $72~^\circ \mathrm{C}$, the transition being reversible on cooling^9–11^. The values of the (complex) relative permittivity of VO$_2$ at any value of the free-space wavelength $\lambda$ are significantly different in its two crystallographic phases. As VO$_2$ is a dissipative insulator (or semiconductor) (I phase) when monoclinic but metallic (M phase) when tetragonal provided that the free-space wavelength $\lambda \gtrsim 1300$ nm, it can be used in reconfigurable metasurfaces in the infrared^12,13^ and terahertz^14,15^ spectral regimes. The capability in switching of functionality in structures comprising tunable materials is often considered from the *multifunctionality* perspective^16–18^. Besides metasurfaces and metamaterials, applications such as smart windows^19^ should be mentioned. Engineering of phase transition of VO$_2$ promises many new applications^20,21^.

The functionality of a structure comprising tunable materials such as VO$_2$ depends on the manner of their deployment in that structure. Commonly, a metasurface is an array of meta-atoms fabricated atop a slab that may be stratified in the thickness direction but is transversely homogeneous. The transverse dimensions of meta-atoms are assumed to be (much) smaller than $\lambda$. The most common way of using a thermally tunable material, e.g. VO$_2$, in a metasurface is in the form of either an electrically thin layer in the stratified slab^22–26^ or a thin grid^27^ or both. For high transmission for both I and M phases of VO$_2$, that material should be used in the meta-atoms whereas the stratified slab should ideally be non-dissipative^28^. Specifically, the incorporation of VO$_2$ pads or inserts in metallic meta-atoms and other hybrid metal-VO$_2$ meta-atoms can facilitate more useful switching scenarios^29–33^, even if the type of functionality remains the same for both phases. But it can be challenging to deposit VO$_2$ components of the meta-atoms because of the commonplace formation of non-stochiometric vanadium oxide (i.e., VO$_{\zeta }$, where $\zeta$ is not the ratio of two small integers)^34,35^ whose thermal-tunability characteristics can differ very significantly from those of stochiometric forms (such as VO$_2$, V$_2$O$_3$, V$_2$O$_5$, V$_4$O$_7$, etc.).

Meta-atoms whose basic functionality is enabled by a dynamically tunable material, but not by a metal or a common effect of a metal and a phase-change material belonging to the same meta-atom (see, e.g., Refs.^29–32^), provide an alternative approach^28,36–39^. Metasurfaces with *thick* VO$_2$ meta-atoms have been recently utilized in the terahertz and near-infrared regimes^12,40–43^. In these metasurfaces, the meta-atoms may be sufficiently thick to display resonances when VO$_2$ is in the M and I phases, but are insufficiently thin to exhibit resonances only for the M phase. As a result, those meta-atoms may have very restricted usefulness for switchable polarization manipulation and related asymmetric transmission (AT). Very recently, thin VO$_2$ meta-atoms with rectangular patches^44–47^ and split rings^48^ have been proposed. Moreover, VO$_2$ patches were used in one structure with VO$_2$ homogeneous layers^46,47^ or grids^44^, in order to achieve a desired functionality switching. For instance, switching between wideband absorption and wideband reflection^46^, between absorption and reflection-mode polarization manipulation^44,45^, and between broadband and multiband absorption^49^ have been demonstrated. In Ref.^48^, VO$_2$ split rings have been proposed for switchable focusing and generation of Airy beams. Such thermally switchable split rings are expected to be particularly suitable also for transmission-mode polarization manipulation. Complementary metasurfaces, i.e., grids of holes in a VO$_2$ layer, have also been suggested^49,50^. Switchable scattering by isolated meta-atoms containing various tunable materials^51–54^ and arrays of hybrid meta-atoms combining conventional dielectric with a phase-change material^55^ should also be mentioned in the context of thermally switchable functionality.

In this paper, we propose unitary (nongradient) metasurfaces comprising arrays of metal-free VO$_2$ (double-)split-ring meta-atoms and metal-free VO$_2$ grids for the purpose of thermal switching between different functionalities in the 0.45–1.35-THz frequency range, with a focus on switching scenarios that involve transmission-mode polarization conversion. One of the aims here is to obtain *switching between (1) polarization conversion as well as related AT and (2) almost-perfect and frequency-independent transmission (as well as other functionalities)*, by switching between the M and I phases of VO$_2$. The metal-free VO$_2$ split rings have not yet been used in such scenarios and may enable new ones. These scenarios become possible by using two states of the meta-atom array: a phase screen^56^ for the I phase of VO$_2$ and a split-ring resonator array for the M phase of VO$_2$. The resulting mechanism exploits the thermal tunability of VO$_2$^2,3^, geometrical features of VO$_2$ meta-atoms and grids proposed here, and specifics of their design that enable transmission and polarization manipulation properties as well as AT^57–59^, which are changed in a desired manner when VO$_2$ thermally transits from the M/I to I/M phase. A particular goal is to investigate the effects of coupling of meta-atom arrays or meta-atom grids that are made of different materials (i.e., VO$_2$ and a metal such as Cu), and their use for functionality switching.

In line with the aims of this study, the meta-atom is chosen to be much thinner than its transverse dimensions, so that resonances appear when VO$_2$ is in the M phase but not in the I phase. Thus, a VO$_2$ metasurface is simply a phase screen^56^ when VO$_2$ is in the I phase, and the same is true for a VO$_2$ grid of proper thickness. This feature differs from our previous work^60^, in which the height of thermally tunable microrods is large enough to enable resonances for the I phase while a phase-screen regime corresponds to an intermediate phase; however, resonances in the I phase are undesirable for our present purpose.

Our preference here is that the metasurfaces operate in the transmission mode while thermal switching of functionality may include polarization manipulation and AT for one of the VO$_2$ phases. However, the scenarios of functionality switching that change the transmission mode into the reflection mode, or vice versa, are also studied. The proposed meta-atoms differ from the hybrid meta-atoms because they never combine VO$_2$ and a metal in one meta-atom. The concept is introduced and numerically validated for metasurfaces representing *cascades* of meta-atom arrays, but we emphasize that design optimization is beyond the scope of this paper. In contrast with the earlier works on polarization manipulation with thin VO$_2$ meta-atoms^44,45,47^, we consider polarization manipulation only in the transmission mode. Also, in contrast with Ref.^32^, where some of AT regimes were studied, we achieve AT without hybrid VO$_2$-metal meta-atoms. In contrast with Ref.^27^, VO$_2$ grids in the proposed designs directly contribute to polarization manipulation and AT. The structures belonging to the two large classes of the polarization-converting metasurfaces capable of AT^59,61^ are examined. Notably, although a single-array metasurface may produce cross-polarized components, at least two coupled arrays are needed to obtain well pronounced AT for linearly polarized (LP) waves.

## Generic metasurface with thin and arbitrary shaped meta-atoms

**Figure 1:**
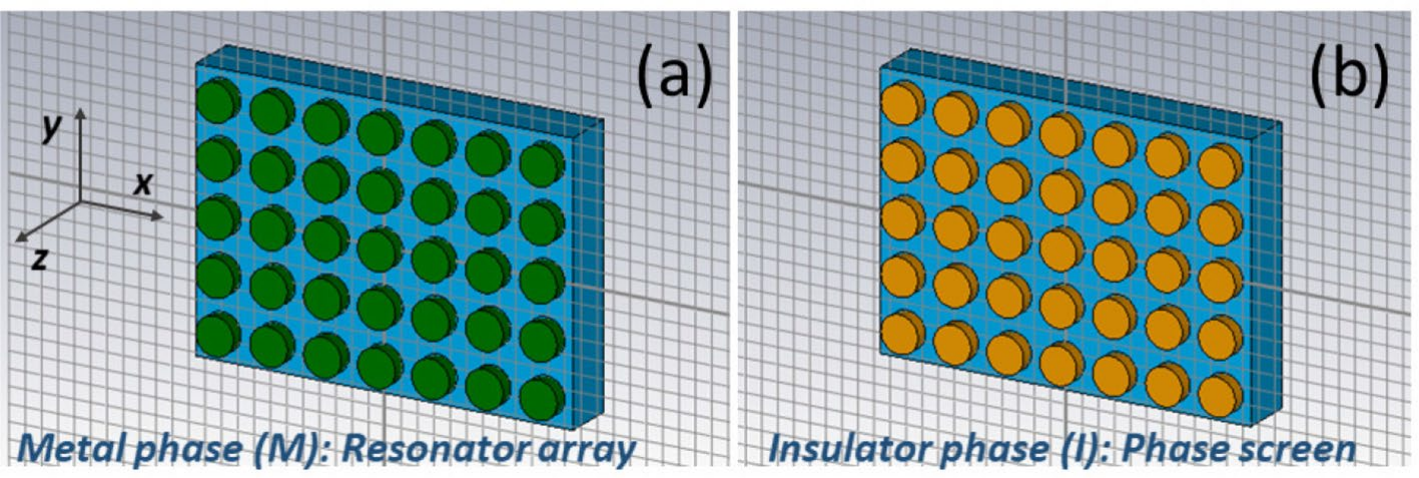
Schematic of a generic single-array metasurface with thin VO$_2$ meta-atoms of arbitrary geometry placed on dielectric substrate (shown in light blue color) when VO$_2$ is in (**a**) the M phase (dark green color) and (**b**) the I phase (orange color). The Cartesian coordinate system is also shown.

A generic single-array metasurface comprising VO$_2$ meta-atoms is illustrated in Fig. 1. It is assumed that the meta-atoms may have rather arbitrary geometry, except that the thickness (here, along the *z* axis) of the VO$_2$ meta-atom should be chosen so that the VO$_2$ array can function as a phase screen for the I phase. For the M phase, the achievable functionality is determined by the geometrical and material properties of the meta-atoms. They are arranged in a biperiodic array in the *xy* plane and are illuminated normally by a LP plane wave. CST Studio Suite^62^ was used to calculate the transmission coefficients $\tau _\mathrm{nm}^Q$ and the reflection coefficients $r_\mathrm{nm}^Q$, where $m\in \left\{ x,y\right\}$ identifies the direction of the electric field of the incident plane wave, $n\in \left\{ x,y\right\}$ identifies the direction of the far-zone transmitted electric field, and the superscript $Q=I$ when VO$_2$ is in the I phase but $Q=M$ when VO$_2$ is in the M phase. The Cartesian unit vectors are denoted by ${\hat{\mathbf{u}}}_x$, ${\hat{\mathbf{u}}}_y$, and ${\hat{\mathbf{u}}}_z$. The coefficients $\tau _\mathrm{nm}^Q$ for front-face and back-face illumination, i.e., for propagation along the $-z$ and $+z$ directions, are additionally superscripted by $\rightarrow$ and $\leftarrow$. Since all materials used are Lorentz reciprocal^63^, $\vert \tau _\mathrm{yy}^\rightarrow \vert =\vert \tau _\mathrm{yy}^\leftarrow \vert$, $\vert \tau _\mathrm{xx}^\rightarrow \vert =\vert \tau _\mathrm{xx}^\leftarrow \vert$, $\vert \tau _\mathrm{xy}^\rightarrow \vert =\vert \tau _\mathrm{yx}^\leftarrow \vert$, and $\vert \tau _\mathrm{yx}^\rightarrow \vert =\vert \tau _\mathrm{xy}^\leftarrow \vert$. Additionally, some kind of geometric symmetry is needed to obtain $\vert \tau _\mathrm{xx}^\rightarrow \vert =\vert \tau _\mathrm{yy}^\rightarrow \vert$ and $\vert \tau _\mathrm{xx}^\leftarrow \vert =\vert \tau _\mathrm{yy}^\leftarrow \vert$. More details can be found in Ref.^57^.

The strongest asymmetry in transmission is expected to be achieved when, say, a forward-propagating incident wave of a specific LP state is fully converted to a transmitted wave of the orthogonal LP state, and the backward-propagating wave of the same LP state is completely reflected; i.e., either

1 $$\begin{aligned} \vert \tau _\mathrm{xy}^\rightarrow \vert \gg \text{ max }(\vert \tau _\mathrm{yx}^\rightarrow \vert ,\vert \tau _\mathrm{xx}^\rightarrow \vert ,\vert \tau _\mathrm{yy}^\rightarrow \vert ) \end{aligned}$$

or

2 $$\begin{aligned} \vert \tau _\mathrm{yx}^\rightarrow \vert \gg \text{ max }(\vert \tau _\mathrm{xy}^\rightarrow \vert ,\vert \tau _\mathrm{xx}^\rightarrow \vert ,\vert \tau _\mathrm{yy}^\rightarrow \vert ). \end{aligned}$$

The relative permittivity of VO$_2$ is given by $\varepsilon _r^{{VO_2}}(\omega )=\varepsilon _\infty - \left( \omega _p^{VO_2}\right) ^2/[\omega (\omega +i/\bar{\tau }^{VO_2})]$, where $\omega =2\pi {f}$ is the angular frequency and *f* is the frequency, $\varepsilon _\infty =9$, $\omega _p^{VO_2}=(\sigma ^{VO_2}/\varepsilon _0\bar{\tau }^{VO_2})^{1/2}$ is the plasma angular frequency, $\varepsilon _0$ is the permittivity of free space, $\bar{\tau }^{VO_2}=2.27$ fs is the relaxation time, and the conductivity $\sigma ^{VO_2}= 40$ S m$^{-1}$ for the I phase ($T=300$ K) and $5\times 10^5$ S m$^{-1}$ for the M phase ($T=400$ K)^64^. For all data reported here, the VO$_2$ thickness *h* was chosen to be realizable with the use of available fabrication techniques^34,35^. In order to enable the phase-screen regime at 1.1 THz with VO$_2$ in the I phase, $h=8.75~\upmu\text{m}$ so that $(2\pi h/\lambda )\left( \varepsilon _r^{{VO_2}}\right) ^{1/2}=0.55+0.022i$. The conductivity of Cu was fixed as $\sigma ^{Cu}=5.96\times 10^7$ S m$^{-1}$.

## Results and discussion

### Switchable metasurfaces with one array of double split rings

**Figure 2:**
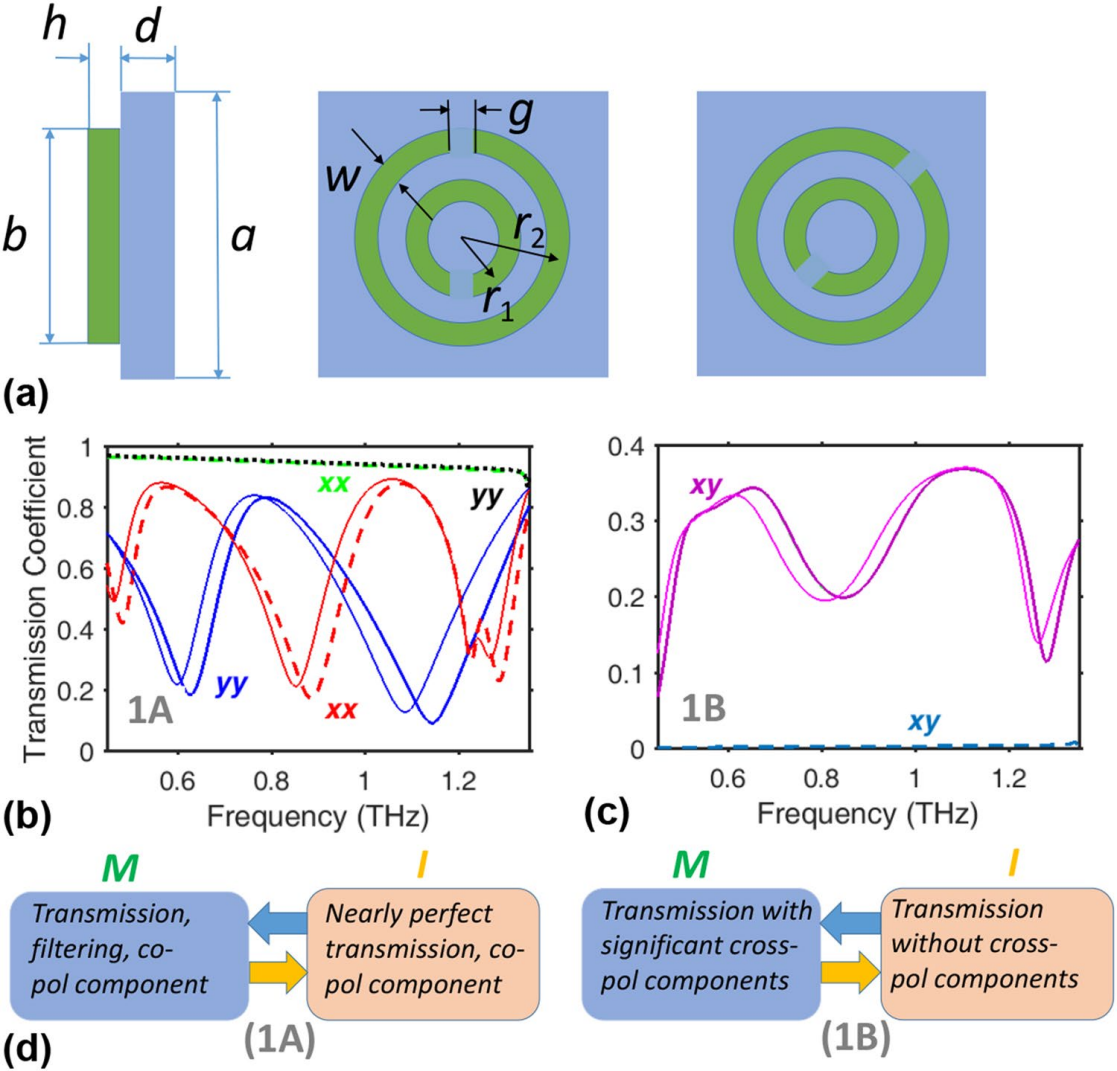
(**a**) From left to right: side cross-section view of the meta-atoms of metasurfaces 1A and 1B, front view of a meta-atom of 1A, and front view of a meta-atom of 1B. Green and blue colors correspond to VO$_2$ DSRs and teflon spacer, respectively. $\vert \tau _\mathrm{nm}^Q\vert$ is plotted as function of *f* for metasurfaces (**b**) 1A and (**c**) 1B, when $a=210~\upmu\text{m}$, $d=19.6875~\upmu \text{m}$, $h=8.75~\upmu \text{m}$, $r_1=44.625~\upmu \text{m}$, $r_2=72.625~\upmu \text{m}$, $w=12.25~\upmu \text{m}$, and $g=14~\upmu\text{m}$. Key: (**b**) $\vert \tau _\mathrm{xx}^M\vert$ (dashed red curve), $\vert \tau _\mathrm{yy}^M\vert$ (solid thicker blue curve), $\vert \tau _\mathrm{xx}^I\vert$ (dash-dotted green curve), and $\vert \tau _\mathrm{yy}^I\vert$ (dotted black curve); $\vert \tau _\mathrm{xx}^I\vert \approx \vert \tau _\mathrm{yy}^I\vert$; (**c**) $\vert \tau _\mathrm{xy}^M\vert$ (solid thicker violet curve) and $\vert \tau _\mathrm{xy}^I\vert$ (dashed blue curve). For comparison, spectral plots of (**b**) $\vert \tau _\mathrm{xx}^M\vert$ (solid thinner blue curve) and $\vert \tau _\mathrm{yy}^M\vert$ (solid thin red curve), and (**c**) $\vert \tau _\mathrm{xy}^M\vert$ (solid thinner violet curve) correspond to $h=2.625~\upmu \text{m}$, and the same remaining parameters. Labels *xx*, *yy*, *xy* are subscripts of $\tau$; they are shown near and by the same color as the corresponding curve(s). (**d**) Left and right panels summarize the functionality switching demonstrated by metasurfaces 1A and 1B, respectively.

Let us begin with the simplest illustration of switching that exploits the nearly complete electromagnetic disappearance of VO$_2$ in the I phase to enable switching for spectral filtering^65^. In Fig. 2, the meta-atom of metasurface 1A comprises two concentric rings of VO$_2$, each with a small air gap *g* aligned parallel to ${\hat{\mathbf{u}}}_y$, on top of a teflon substrate of relative permittivity equal to 2.1 and thickness denoted by *d*. Whereas the meta-atom is of transverse dimensions $a\times {a}$, the thickness of the double split-ring (DSR) structure along the *z* axis is denoted by *h*. The inner split ring has mean radius $r_1$ and width *w*. The outer split ring has outer diameter $b<a$, width *w*, and mean radius $r_2=(b-w)/2$.

Representative spectra of the magnitudes of the co-polarized transmission coefficients $\vert \tau _{xx}^M\vert$, $\vert \tau _{yy}^M\vert$, $\vert \tau _{xx}^I\vert$, and $\vert \tau _{yy}^I\vert$ are presented in Fig. 2b,c. Whereas frequency-selective transmission occurs when VO$_2$ is in the M phase, nearly frequency-independent and high transmission occurs for $f\in [0.45,1.35]$ THz when VO$_2$ is in the I phase. In the latter case, $\varepsilon _r^{{VO_2}}=8.99+0.72i$ at $f=1$ THz so that VO$_2$ cannot be said to be in the vacuum state (i.e., $\text{ Re }\varepsilon _r\ne 1$ on contrary to Ref.^60^), but metasurface 1A functions as a phase screen with a relatively slight attenuation.

When VO$_2$ is in the M phase, transmission through the metasurface 1A depends on the polarization state of the incident plane wave. As can be observed in Fig. 2b, the minimums and maximums of $\vert \tau _{mm}\vert$ have different spectral locations for $\mathbf{E} _\mathrm{inc}\parallel {\hat{\mathbf{u}}}_x$ (i.e., $m=x$) than for $\mathbf{E} _\mathrm{inc}\parallel {\hat{\mathbf{u}}}_y$ (i.e., $m=y$). These spectral locations can be shifted by changing *h*. Whereas $\text{ max }~\vert \tau _{xx}^M\vert >0.8$ but $\text{ min }~\vert \tau _{yy}^M\vert <0.2$ in some spectral regimes, $\text{ max }~\vert \tau _{yy}^M\vert >0.8$ but $\text{ min }~\vert \tau _{xx}^M\vert <0.2$ in other spectral regimes. In contrast, $\vert \tau _{xx}^I\vert \simeq \vert \tau _{yy}^I\vert >0.87$ for $f\in [0.45,1.35]$ THz. Phase change may lead to a strong difference in transmission strength ($\vert \tau ^I\vert ^2/\vert\tau ^M\vert ^2> 50$) in some spectral regimes, but a weak difference ($\vert \tau ^I\vert ^2/\vert\tau ^M\vert ^2\le 1.2$) in others.

Rotation of a single-array metasurface, such as metasurface 1A, is a simple way to obtain significant cross-polarized transmission when the incident plane wave is linearly polarized^66^. To demonstrate the capability of VO$_2$ DSR structures for cross-polarized transmission, we rotated metasurface 1A about the *z* axis by $45^\circ$ to obtain metasurface 1B. Figure 2c presents spectra of the transmission coefficients $\vert \tau _{xy}\vert =\vert \tau _{yx}\vert$ of metasurface 1B. The results are presented for the both M and I phases of VO$_2$. Switching between nearly zero (OFF state, I phase) and moderately efficient (ON state, M phase) cross-polarized transmission is achieved. Manipulation of LP state by a metasurface has been often considered in connection with AT^57,59^, when transmission coefficients depend on which one of the faces of the metasurface is being illuminated. The single-array metasurfaces such as 1B are not capable of exhibiting AT in the case of LP incidence, regardless of the crystallographic phase of VO$_2$.

### Switchable metasurfaces with coupled arrays of double split rings

**Figure 3:**
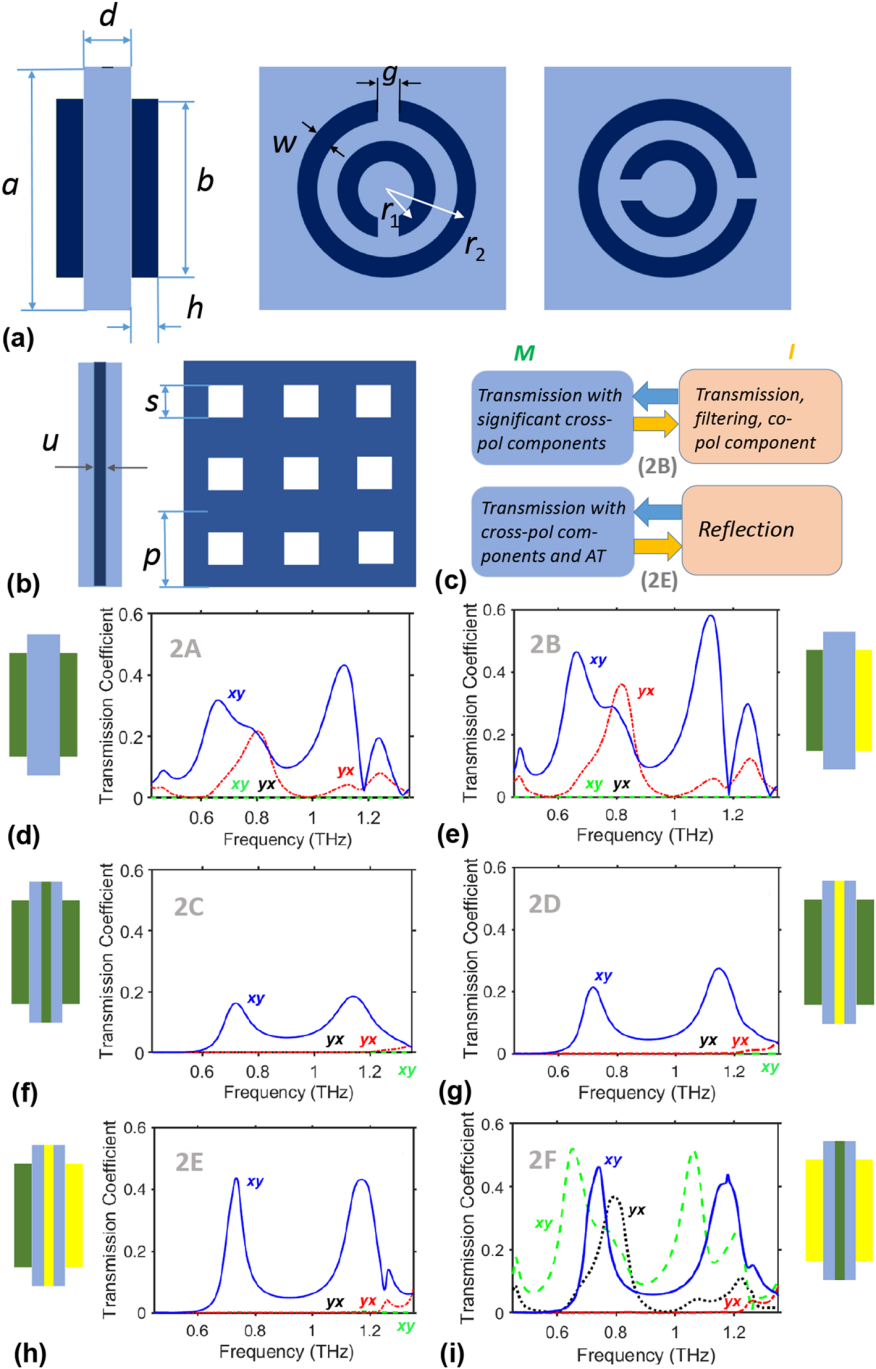
(**a**) From left to right: side cross-section view of a unit cell of metasurface 2A, 2B with a dielectric spacer, and front and back views of a unit cell of metasurfaces 2A–2F. Dark blue and light blue colors correspond here to DSRs and spacers, respectively. The DSR on the front face is made of VO$_2$ (2A–2E) or Cu (2F), and the DSR on the back face is made of VO$_2$ (2A, 2C, 2D) or Cu (2B, 2E, 2F). (**b**) Side cross-section and mid-cross-section views of the spacer of thickness *d*, which comprises either a Cu grid (2D, 2E) or a VO$_2$ grid (2C, 2F) in the middle. (**c**) Examples of the functionality switching vs. VO$_2$ phase for metasurfaces 2B and 2E. $\vert \tau _\mathrm{nm}^Q\vert$ vs. *f* is plotted for metasurfaces (**d**) 2A, (**e**) 2B, (**f**) 2C, (**g**) 2D, (**h**) 2E, and (**i**) 2F, at $a=210~\upmu \text{m}$, $d=19.69~\upmu \text{m}$, $h=8.75~\upmu \text{m}$, $r_1=44.63~\upmu \text{m}$, $r_2=72.63~\upmu \text{m}$, $w=12.25~\upmu \text{m}$, $g=14~\upmu \text{m}$, $p=70~\upmu \text{m}$, $s=35~\upmu \text{m}$, $u=3.94~\upmu \text{m}$. Key: $\vert \tau _\mathrm{xy}^{M\rightarrow }\vert =\vert \tau _\mathrm{yx}^{M\leftarrow }\vert$ (solid blue curves), $\vert \tau _\mathrm{yx}^{M\rightarrow }\vert =\vert \tau _\mathrm{xy}^{M\leftarrow }\vert$ (dash-dotted red curves), $\vert \tau _\mathrm{xy}^{I\rightarrow }\vert =\vert \tau _\mathrm{yx}^{I\leftarrow }\vert$ (dashed green curves), and $\vert \tau _\mathrm{yx}^{I\rightarrow }\vert =\vert \tau _\mathrm{xy}^{I\leftarrow }\vert$ (dotted black curves); $\vert \tau _\mathrm{xy}^{I\rightarrow }\vert \approx 0$ and $\vert \tau _\mathrm{yx}^{I\rightarrow }\vert \approx 0$ in (**d**–**h**). Labels *xy* and *yx* are shown near and by the same color as the corresponding curve(s) that are the subscripts of $\tau ^{\rightarrow }$. The insets in (**d**–**i**) present schematics of composition of a unit cell of the metasurface, in accordance with (**a**,**b**); dark green and yellow colors stand for VO$_2$ and Cu components, respectively, and blue color stands for the teflon spacers.

To demonstrate polarization manipulation and, in particular, asymmetry of the cross-polarized components (i.e., $\vert \tau _\mathrm{xy}^{M\rightarrow }\vert \ne \vert \tau _\mathrm{xy}^{M\leftarrow }\vert$) due to metasurfaces comprising metal-free VO$_2$ meta-atoms and metal-free VO$_2$ grids, we first consider the ones with both front and back faces having DSRs in their unit cells; see Fig. 3. Each meta-atom in the *double-array* metasurface 2A has a VO$_2$ DSR on the front face and its rotated version on the back face, with a teflon ($\varepsilon _r=2.1$) spacer between the front and back DSRs. Geometrically similar metasurfaces but with metallic resonators have been previously investigated for diodelike AT^57,58^. For metasurface 2A, cross-polarized transmission is expected to appear due to the coupling of two metal-free arrays in the M-phase of VO$_2$.

Representative spectra of $\vert \tau _\mathrm{xy}^{Q\rightarrow }\vert =\vert \tau _\mathrm{yx}^{Q\leftarrow }\vert$ and $\vert \tau _\mathrm{yx}^{Q\rightarrow }\vert =\vert \tau _\mathrm{xy}^{Q\leftarrow }\vert$ are shown in Fig. 3d. Here, we obtain $\vert \tau _\mathrm{xy}^{M\rightarrow }\vert =0.32$ but $\vert \tau _\mathrm{xy}^{M\leftarrow }\vert =0.04$ at $f=0.66$ THz, and $\vert \tau _\mathrm{xy}^{M\rightarrow }\vert =0.44$ but $\vert \tau _\mathrm{xy}^{M\leftarrow }\vert =0.04$ at $f=1.125$ THz, while $\vert \tau _\mathrm{xx}^{M\rightarrow }\vert =\vert \tau _\mathrm{yy}^{M\rightarrow }\vert =0.44$ and 0.3, respectively. Hence, there is quite a good coupling between the M-phase VO$_2$ DSRs, although metallic components are not used. Cross-polarized transmission does not occur in the I phase of VO$_2$, because the VO$_2$ arrays function as phase screens. Thus, cross-polarized transmission can be switched ON/OFF, i.e., the VO$_2$ phase change delivers a change between (i) a spectrally selective transmission regime with cross-polarized components and (ii) highly efficient and frequency-independent transmission without such components.

Intuitively, the efficiency can be increased by decreasing the overall losses and/or increasing the overall conduction capability. This is demonstrated by metasurface 2B, which differs from 2A only in that the DSRs on the back face are made of Cu. Therefore, in contrast with many polarization-manipulating metasurfaces^57–59^, metasurface 2B contains two *dissimilar* arrays of DSRs. Despite the significant difference between the DSR arrays in terms of conductivity, they are well coupled and deliver moderately high cross-polarized transmission, when VO$_2$ is in the M phase. The transmission spectra presented in Fig. 3e have two distinct spectral regimes for AT: one with $\vert \tau _\mathrm{xy}^{M\rightarrow }\vert =0.46$ and $\vert \tau _\mathrm{xy}^{M\leftarrow }\vert =0.06$ at $f=0.665$ THz and the other with $\vert \tau _\mathrm{xy}^{M\rightarrow }\vert =0.58$ and $\vert \tau _\mathrm{xy}^{M\leftarrow }\vert =0.057$ at $f=1.125$ THz, both with at least 50-fold difference between $\vert \tau _\mathrm{xy}^{M\rightarrow }\vert ^2$ and $\vert \tau _\mathrm{yx}^{M\rightarrow }\vert ^2$. The VO$_2$ array functions as a phase screen for the I phase, so that metasurface 2A may transmit significantly but without cross-polarized components. Thus, cross-polarized transmission can be switched ON/OFF. However, co-polarized transmission remains strong in the M phase; e.g., (a) $\vert \tau _\mathrm{xx}^{M\rightarrow }\vert =0.35$ and $\vert \tau _\mathrm{yy}^{M\rightarrow }\vert =0.47$ at $f=0.665$ THz, and (b) $\vert \tau _\mathrm{xx}^{M\rightarrow }\vert =0.28$ and $\vert \tau _\mathrm{yy}^{M\rightarrow }\vert =0.3$ at $f=1.125$ THz. For case (b), the ratio of the overall forward-to-backward transmission intensities is about 4.3 when $\mathbf{E} _\mathrm{inc}\parallel {\hat{\mathbf{u}}}_x$ and 3.7 when $\mathbf{E} _\mathrm{inc}\parallel {\hat{\mathbf{u}}}_y$. This indicates a relatively low contrast for AT, so that a different design is needed to suppress co-polarized transmission, per conditions set by Eqs. (1) and (2). In terms of functionality switching, metasurface 2B is similar to 2A, because the transmission mode is conserved for both the M and I phases. However, transmission in the I phase now depends on the frequency.

To ensure suppression of both co-polarized and one of the two cross-polarized components, in line with Eqs. (1) and (2), we designed the *triple-array* metasurface 2C to have VO$_2$ DSRs on the front face and rotated VO$_2$ DSRs on the back face, with a VO$_2$ grid inserted in the middle of the spacer, i.e., similarly to the Cu grid in Ref.^59^. Representative transmission spectra are shown in Fig. 3f. Now we have $\vert \tau _\mathrm{xy}^{M\rightarrow }\vert \gg \vert \tau _\mathrm{yx}^{M\rightarrow }\vert$ and $\vert \tau _\mathrm{xy}^{M\rightarrow }\vert \gg \mathrm{max}{(\vert \tau _\mathrm{xx}^{M\rightarrow }\vert ,\vert \tau _\mathrm{yy}^{M\rightarrow }\vert )}$, as desired. Metasurface 2C works as an AT device in the M phase and somewhat as a phase screen in the I phase. However, the peak values of $\vert \tau _\mathrm{xy}^{M\rightarrow }\vert$ are relatively low, because of high ohmic losses and quite strong reflection.

In order to enhance cross-polarized transmission, we replaced the VO$_2$ grid in metasurface 2C by a geometrically identical Cu grid. The transmission spectra of the resulting metasurface 2D are presented in Fig. 3g. Now, we have $\vert \tau _\mathrm{xy}^{M\rightarrow }\vert =0.21$ instead of 0.16 at $f=0.72$ THz and $\vert \tau _\mathrm{xy}^{M\rightarrow }\vert =0.27$ instead of 0.185 near $f=1.14$ THz. Metasurface 2D exhibits AT when VO$_2$ is in the M phase but functions as an almost perfect reflector when VO$_2$ is in the I phase. Hence, in terms of functionality, it is not identical to metasurface 2C. As an example of AT efficiency in the M phase, $\vert \tau ^{M\rightarrow }\vert ^2/\vert \tau ^{M\leftarrow }\vert ^2\approx 270$ and 28 at $f=0.72$ THz and 1.14 THz, respectively.

The *triple-array* metasurface 2E was designed to further enhance AT efficiency while preserving the capability of ON/OFF switching. It has VO$_2$ DSRs on the front face, rotated Cu DSRs on the back face, and a Cu grid at the middle. Transmission spectra are presented in Fig. 3h. Compared to metasurface 2D, $\vert \tau _\mathrm{xy}^{M\rightarrow }\vert$ is now as twice as large. An efficient coupling of two different arrays of DSRs in metasurface 2E for VO$_2$ in the M phase is evident. For VO$_2$ in the I phase, metasurface 2E shows high reflection for a larger part of the considered frequency range but has windows of small-to-moderate co-polarized transmission.

Figure 3i presents some results for metasurface 2F, in which the grid is made of VO$_2$ whereas all DSRs are made of Cu. When VO$_2$ is in the M phase, AT is strong with the unwanted transmission components suppressed. In particular, $\vert \tau _\mathrm{xy}^{M\rightarrow }\vert =0.465$ at $f=0.74$ THz and $\vert \tau _\mathrm{xy}^{M\rightarrow }\vert =0.44$ at $f=1.179$ THz, but $\vert \tau _\mathrm{xx}^{M\rightarrow }\vert \le {0.06}$ and $\vert \tau _\mathrm{yx}^M{\rightarrow }\vert \le {0.01}$. When VO$_2$ is in the I phase, the grid functions as a phase screen and both $\vert \tau _\mathrm{xx}^{I\rightarrow }\vert$ and $\vert \tau _\mathrm{xy}^{I\rightarrow }\vert$ are significant; e.g., $\vert \tau _\mathrm{xx}^{I\rightarrow }\vert =\vert \tau _\mathrm{yy}^{I\rightarrow }\vert =0.37$ and $\vert \tau _\mathrm{xy}^{I\rightarrow }\vert =0.52$ at $f=0.65$ THz while $\vert \tau _\mathrm{yx}^{I\rightarrow }\vert \le {0.05}$. To compare, $\vert \tau _\mathrm{xx}^{I\rightarrow }\vert =\vert \tau _\mathrm{yy}^{I\rightarrow }\vert =0.22$ and $\vert \tau _\mathrm{xy}^{I\rightarrow }\vert =0.52$ at $f=1.065$ THz, whereas $\vert \tau ^{I\rightarrow }\vert ^2/\vert \tau ^{I\leftarrow }\vert ^2=5.58$ (the case of a relatively weak asymmetry in transmisson). Roughly speaking, whereas metasurface 2F for the M phase is similar to 2E for the M phase, 2F for the I phase is similar to 2B for the M phase. In terms of functionality, the phase change of VO$_2$ results here in switching between AT for the M phase and spectrally selective transmission with significant co- and cross-polarized components for the I phase.

The obtained results show that high-contrast switchable AT can be achieved (at least with moderate efficiency) in the structures comprising two coupled VO$_2$ DSR arrays, whereas transmission mode is conserved for both the M and I phases of VO$_2$. Using only one VO$_2$ array and coupling of VO$_2$ and Cu arrays, we can achieve higher transmittance in the AT regime when VO$_2$ is the M phase, but at the price of abandoning the transmission mode for the I phase. It is worth noting that using two different arrays in one structure (such as for the metasurfaces 2B and 2E) generally leads to $\vert \tau _\mathrm{xx}^{M\rightarrow }\vert \ne \vert \tau _\mathrm{yy}^{M\rightarrow }\vert$ and $\vert \tau _\mathrm{xx}^{M\leftarrow }\vert \ne \vert \tau _\mathrm{yy}^{M\leftarrow }\vert$. However, for the proposed metasurfaces, selection of one or the other LP state for the incident wave does not lead to the principal difference in the functionality-switching scenarios.

### Switchable few-layer metasurfaces with one split-ring array

Next, we present a set of few-array metasurfaces designed for switchable AT enabled by polarization conversion. In contrast with “Switchable metasurfaces with coupled arrays of double split rings”, the metasurfaces studied here comprise only one array of subwavelength split rings. Notably, few-array non-switchable metasurfaces have been studied earlier that are either capable or incapable of polarization manipulation and AT^61,67–72^. Our goal here is to obtain high-efficiency *broadband* polarization conversion and related AT in a switching scenario. The polarization-converting (but non-switchable) structures from Refs.^61,73^ were taken as the pre-prototypes.

**Figure 4:**
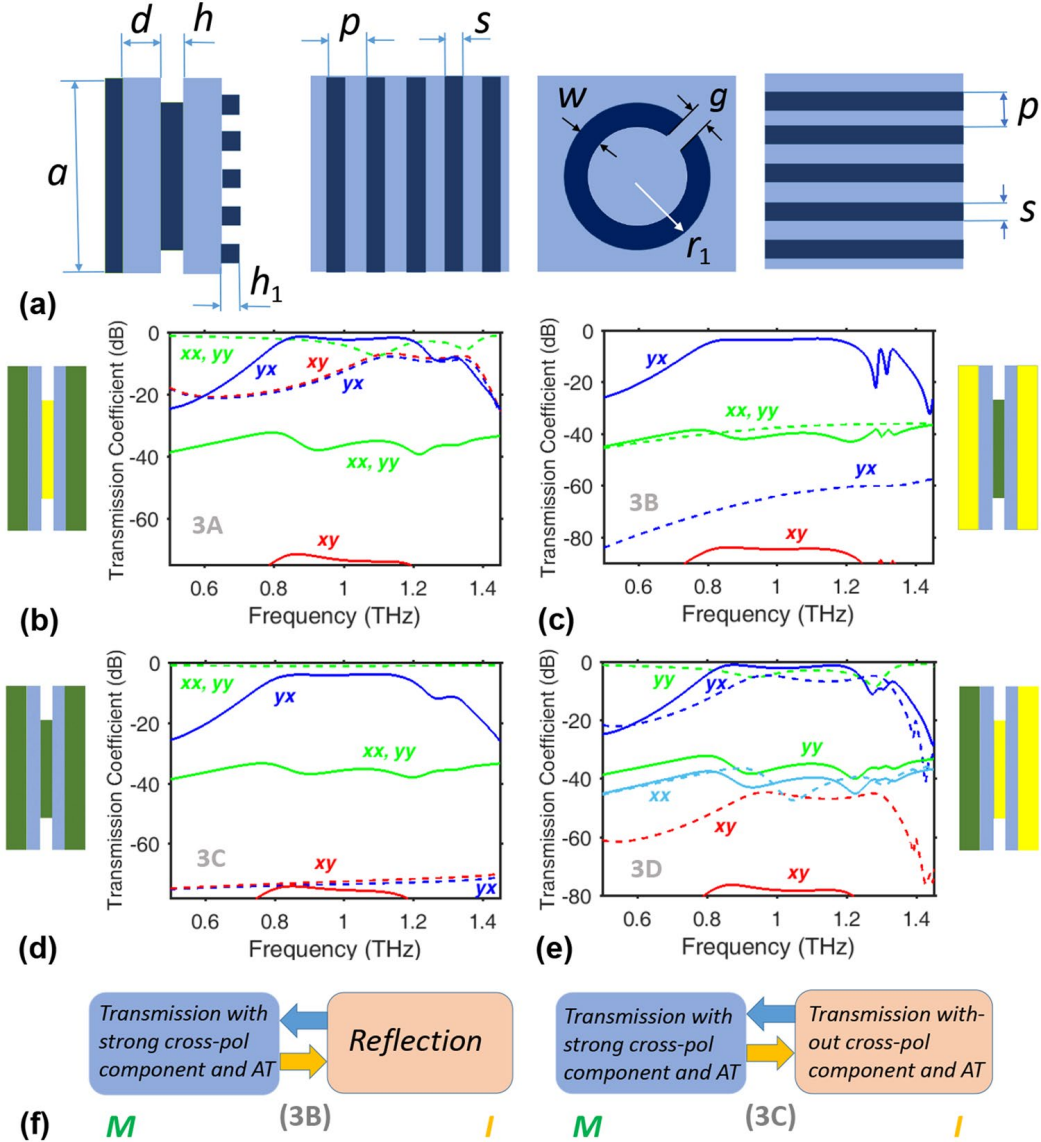
(**a**) From left to right: side-cross-section view, front view, mid-cross-section view, and back view of a unit cell of metasurfaces 3A–3D. Dark blue color corresponds to SSRs and strip grids, and light blue color does to dielectric spacers. The strips on the front face are made of VO$_2$ (3A, 3C, 3D) or Cu (3B), the SSR are made of VO$_2$ (3B, 3C) or Cu (3A, 3D), and the strips on the back face are made of VO$_2$ (3A, 3C) or Cu (3B, 3D). $\vert \tau _\mathrm{nm}^Q\vert$ vs. *f* is plotted for metasurfaces (**b**) 3A, (**c**) 3B, (**d**) 3C, and (**e**) 3D, when $a=165~\upmu \text{m}$, $d=18.90~\upmu \text{m}$, $p=13.75~\upmu \text{m}$, $s=5.50~\upmu \text{m}$, $h=h_1=3.78~\upmu \text{m}$, $r_1=37.81~\upmu \text{m}$, and $g=w=6.89~\upmu \text{m}$. Key: $\vert \tau ^M_\mathrm{xx}\vert$ (solid green curves in (**b**–**d**) and solid light-blue curve in (**e**)), $\vert \tau ^M_\mathrm{yy}\vert$ (solid green curves), $\vert \tau ^I_\mathrm{xx}\vert$ (dashed green curves in (**b**–**d**) and dashed light-blue curve in (**e**)), $\vert \tau ^I_\mathrm{yy}\vert$ (dashed green curves), $\vert \tau _\mathrm{xy}^{M\rightarrow }\vert =\vert \tau _\mathrm{yx}^{M\leftarrow }\vert$ (solid red curves), $\vert \tau _\mathrm{yx}^{M\rightarrow }\vert =\vert \tau _\mathrm{xy}^{M\leftarrow }\vert$ (solid dark-blue curves), $\vert \tau _\mathrm{xy}^{I\rightarrow }\vert =\vert \tau _\mathrm{yx}^{I\leftarrow }\vert$ (dashed red curves), and $\vert \tau _\mathrm{yx}^{I\rightarrow }\vert =\vert \tau _\mathrm{xy}^{I\leftarrow }\vert$ (dashed dark-blue curves). Labels *xy*, *yx*, *xx*, and *yy* are shown near and by the same color as the corresponding curve(s) that are the subscripts of $\tau ^{\rightarrow }$. The insets in (**b**–**e**) present schematics of composition of a unit cell of the metasurface, in accordance with (**a**); dark green and yellow stand for VO$_2$ and Cu components, respectively, and blue stands for the spacers. (**f**) Examples of the functionality switching for metasurfaces 3B (left panel) and 3C (right panel).

Each unit cell of the triple-array metasurfaces 3A–3D shown in Fig. 4 has a front grid of strips parallel to the *y* axis, a single split ring (SSR) in the middle with a gap aligned parallel at $45^{\circ }$ to both the *x* and *y* axes, and a back grid of strips parallel to the *x* axis. Any two consecutive arrays are separated by a silica ($\varepsilon _r=2.25$) spacer. The strips on the front face are made of VO$_2$ (3A, 3C, 3D) or Cu (3B), the SSR is made of VO$_2$ (3B, 3C) or Cu (3A, 3D), and the strips on the back face are made of VO$_2$ (3A, 3C) or Cu (3B, 3D). Transmission spectra are presented in Fig. 4, first of all, to demonstrate the capability of exhibiting AT.

As shown in Fig. 4b for metasurface 3A, the M phase of VO$_2$ delivers strong polarization conversion and AT, since all three arrays work as metallic arrays. Both grids operate as highly transmissive phase screens when VO$_2$ is in the I phase, so that the Cu SSR array by itself enables the cross-polarized components but without AT. For $f\in [0.8,1]$ THz, switching between the M and I phases of VO$_2$ leads to the functionality switching between transmission-mode polarization conversion (and related AT) and spectrally selective transmission with the dominant co-polarized component. For instance, when VO$_2$ is in the M phase, $\vert \tau ^{M\rightarrow }\vert ^2/\vert \tau ^{M\leftarrow }\vert ^2\approx 3\times {10^3}$ and $\vert \tau _\mathrm{yx}^{M\rightarrow }\vert =0.87$ at $f=0.88$ THz. Interestingly, at $f=1.3$ THz, $\vert \tau _\mathrm{yx}^{\rightarrow }\vert$ is weakly sensitive to a phase change, but $\vert \tau _\mathrm{xy}^{\rightarrow }\vert$ is strongly sensitive. Comparable co- and cross-polarized components occur for the I phase at 1.15 THz. Thus, diverse scenarios can be achieved with metasurface 3A functioning in the transmission mode for both phases of VO$_2$.

VO$_2$ and Cu are interchanged in metasurface 3B. Figure 4c shows that strong polarization conversion and AT are achieved when VO$_2$ is in the M phase, so that $\vert \tau _\mathrm{yx}^{M\rightarrow }\vert \gg \vert \tau _\mathrm{yx}^{M\leftarrow }\vert$ for the entire frequency range, and $\vert \tau _\mathrm{yx}^{M\rightarrow }\vert \gg \vert \tau _\mathrm{xx}^{M\rightarrow }\vert$ for most of it. The VO$_2$ SSR array functions as a transmissive phase screen in the I phase, i.e., without AT. In terms of functionality, we obtain switching between transmission-mode polarization conversion and AT for the M phase and reflection in the I phase. This metasurface is well suited to function as an ON/OFF switchable diodelike AT device, but the transmission mode is not conserved while switching.

Both strip grids and the SSR arrays in metasurface 3C function as highly transmissive phase screens when VO$_2$ is in the I phase, but as metal arrays when it is in the M phase. As a result, we obtain strong polarization conversion and related AT for the M phase and the almost perfect and *f*-independent transmission without polarization conversion for the I phase for $0.65<f<1.05$ THz in Fig. 4d. Thus, broadband ON/OFF switching of AT can be obtained in the transmission mode. For example, when VO$_2$ is in the M phase, $\vert \tau ^{M\rightarrow }\vert ^2/\vert \tau ^{M\leftarrow }\vert ^2=2.85\times {10^3}$ and $\vert \tau _\mathrm{yx}^{M\rightarrow }\vert =0.64$ at $f=0.88$ THz.

**Table 1:** Comparison of metasurfaces 3A-3D in terms of selected AT characteristics and the basic scenarios of functionality switching, according to Fig. 4.

Metasurface design	Operating band of AT with VO$_2$ in M phase, in terms of $|\tau _{yx}^{M\rightarrow }|$	AT contrast	Difference in $|\tau _{yx}^{\rightarrow }|$ within AT band from M to I phase change of VO$_2$	Functionality switching from M/I to I/M phase change of VO$_2$
3A	> $-3$ dB for $0.82<f<1.2$ THz	> 28 dB	> 10 dB for $0.82<f<0.97$ THz	AT to/from transmission with stronger co- and weaker cross-pol components
3B	> $-3.5$ dB for $0.83<f<1.17$ THz	> 35 dB	> 30 dB for $0.83<f<1.17$ THz	AT to/from dominant reflection
3C	> $-4$ dB for $0.84<f<1.14$ THz	> 31 dB	> 65 dB for $0.84<f<1.17$ THz	AT to/from transmission with nearly perfect, frequency independent co-pol transmission component
3D	> $-1.86$ dB for $0.82<f<1.2$ THz	> 33 dB	8.4 dB for $f\approx 0.85$ THz	One AT regime to/from another for one polarization, and reflection to/from dominant co-pol transmission for the other

Only the strip grid on the front face is made of VO$_2$ in metasurface 3D, the other two arrays being made of Cu. Similarly to Fig. 3g, there are two geometrically identical arrays (strip grids, here), which are rotated with respect to each other and made of different materials. The VO$_2$ strip grid is a transmissive phase screen when VO$_2$ is in the I phase, but a metallic grid otherwise. In Fig. 4e, $\vert \tau _\mathrm{yx}^{M\rightarrow }\vert \gg \vert \tau _\mathrm{xy}^{M\rightarrow }\vert$, $\vert \tau _\mathrm{yx}^{M\rightarrow }\vert \gg \vert \tau _\mathrm{xx}^{M\rightarrow }\vert$, and $\vert \tau _\mathrm{yx}^{M\rightarrow }\vert \gg \vert \tau _\mathrm{yy}^{M\rightarrow }\vert$, when VO$_2$ is in the M phase; otherwise, $\vert \tau _\mathrm{yx}^{I\rightarrow }\vert \gg \vert \tau _\mathrm{xy}^{I\rightarrow }\vert$ and $\vert \tau _\mathrm{yx}^{I\rightarrow }\vert \gg \vert \tau _\mathrm{xx}^{I\rightarrow }\vert$, but $\vert \tau _\mathrm{yx}^{I\rightarrow }\vert \propto \vert \tau _\mathrm{yy}^{I\rightarrow }\vert$. In particular, $\vert \tau _\mathrm{yx}^{M\rightarrow }\vert =0.91$ and $\left[ \vert \tau _\mathrm{xx}^{M\rightarrow }\vert ^2+\vert \tau _\mathrm{yx}^{M\rightarrow }\vert ^2 \right] / \left[ \vert \tau _\mathrm{xx}^{M\leftarrow }\vert ^2+\vert \tau _\mathrm{yx}^{M\leftarrow }\vert ^2 \right] =9.78\times {10^3}$ for $f=0.88$ THz (a maximum of $\vert \tau _\mathrm{yx}^{M\rightarrow }\vert$), $\vert \tau _\mathrm{yx}^{I\rightarrow }\vert =0.41$ and $\left[ \vert \tau _\mathrm{xx}^{I\rightarrow }\vert ^2+\vert \tau _\mathrm{yx}^{I\rightarrow }\vert ^2 \right] / \left[ \vert \tau _\mathrm{xx}^{I\leftarrow }\vert ^2+\vert \tau _\mathrm{yx}^{I\leftarrow }\vert ^2 \right] \approx 700$ for $f=0.88$ THz, and $\vert \tau _\mathrm{yx}^{I\rightarrow }\vert =0.59$ and $\left[ \vert \tau _\mathrm{xx}^{I\rightarrow }\vert ^2+\vert \tau _\mathrm{yx}^{I\rightarrow }\vert ^2 \right] / \left[ \vert \tau _\mathrm{xx}^{I\leftarrow }\vert ^2+\vert \tau _\mathrm{yx}^{I\leftarrow }\vert ^2 \right] =3.48\times {10^3}$ for $f=0.97$ THz (a maximum of $\vert \tau _\mathrm{yx}^{I\rightarrow }\vert$). Note that $\vert \tau _\mathrm{yx}^{M\rightarrow }\vert >\vert \tau _\mathrm{yx}^{I\rightarrow }\vert$ when $0.8<f<1.24$ THz, but $\vert \tau _\mathrm{yx}^{M\rightarrow }\vert <\vert \tau _\mathrm{yx}^{I\rightarrow }\vert$ near $f=1.27$ THz. At $f=1.24$ THz and 1.32 THz, $\vert \tau _\mathrm{yx}^{\rightarrow }\vert$ is insensitive to the crystallographic phase of VO$_2$. The obtained switching scenarios strongly depend on the polarization state of the incident wave. When $\mathbf{E} _\mathrm{inc}\parallel {\hat{\mathbf{u}}}_x$, switching between two AT regimes with strong cross-polarized and weak co-polarized transmission components can be achieved by thermally changing the crystallographic phase of VO$_2$. When $\mathbf{E} _\mathrm{inc}\parallel {\hat{\mathbf{u}}}_y$, switching between strong co-polarized transmission for the I phase of VO$_2$ and strong reflection for the M phase of VO$_2$ is obtained. However, in the contrast with metasurfaces 3A and 3C, nearly perfect frequency- and polarization independent co-polarized transmission cannot be achieved for 3D within the used frequency range.

The capabilities of metasurfaces 3A-3D for AT are compared in Table 1 by using the quantities plotted in Fig. 4. AT contrast is evaluated as the difference (in dB) between $|\tau _{yx}^{M\rightarrow }|$ and $\text{ max }(|\tau _{yx}^{M\leftarrow }|, |\tau _{yx}^{M\leftarrow }|)$, provided that $|\tau _{yx}^{M\rightarrow }|\gg |\tau _{xx}^{M\rightarrow }|$; see Eqs. (1) and (2). Since the switching of AT band is enabled by one of the cross-polarized transmission components, it is quantified here in terms of the difference (in dB) between $|\tau _{yx}^{M\rightarrow }|$ and $|\tau _{yx}^{I\rightarrow }|$.

A remarkable result observed in Fig. 4 is that the highly efficient, switchable, broadband polarization conversion and AT can be obtained in the transmission mode for both the M and I phases of VO$_2$. In contrast with Refs.^29–31^, placing VO$_2$ pads/inserts into subwavelength metallic resonators is unnecessary. Moreover, the SSRs can be made of Cu, while only the grids are made of VO$_2$, to enable switching while operating in the transmission mode. One strip grid can be made of VO$_2$ in order to obtain a specific switching between two directionally selective regimes by thermally changing the crystallographic phase of VO$_2$. Note that the VO$_2$ grids in M phase directly contribute to polarization manipulation for metasurfaces 3A, 3C, and 3D, so that their role is not restricted to tunability.

## Discussion

To summarize, we have proposed and numerically validated that few-layer metasurfaces comprising electrically thin metal-free meta-atoms and grids made of VO$_2$ are capable of polarization manipulation and asymmetric transmission. The advantages of using a thermally tunable material, such VO$_2$, include that: (a) the temperature-range required for extreme ON/OFF switching is relatively narrow and (b) no biasing circuit is needed. Only a simple heater is needed on-site. We have demonstrated that the thermal transition from the M/I to the I/M phase of VO$_2$ may result in extreme functional reconfiguration, while either conserving the transmission mode of operation or changing it for the reflection mode.

A key feature is that the arrays of meta-atoms and/or grids made of VO$_2$ function as highly transmissive phase screens for VO$_2$ in the I phase. Metasurfaces comprising designed meta-atoms and grids are capable in diverse scenarios of switchable polarization conversion and AT. In particular, triple-array metasurfaces comprising one SSR array and two strip grids, or two DSR arrays and one grid, may enable switchable polarization conversion and AT, provided that VO$_2$ components are properly incorporated in the device. To operate in the transmission mode for both phases of VO$_2$ and have AT for one of the two phases, (a) all SSR/DSR arrays and grids, or (b) the central grid, or (c) grids on both faces should be made of VO$_2$. The two DSR arrays can be well coupled even if one of them is made of VO$_2$ and the other of a metal, i.e., when the difference in DSR conductivities is significant; indeed, the smaller conductivity of VO$_2$ in the M phase, as compared to that of the metal, does not lead to the disappearance of switchable polarization conversion and AT. The same is true for triple-array metasurfaces with one face grid made of VO$_2$ and the other face grid made of Cu. Significant dependence of the co-polarized transmission on the polarization state of the incident wave may enable an additional degree of freedom in functionality switching. The proposed concept and sample designs constitute a perfect platform to design new switchable, multifunctional, polarization-manipulating, and diodelike THz devices.

Although considerable theoretical literature on metasurfaces containing VO$_2$ exists, idealized constitutive parameters are used therein. As stated earlier, it is challenging to deposit pure VO$_2$ because it is difficult to avoid the formation of polycrystalline vanadium oxide (i.e., $\text{ VO}_\mathrm{\zeta }$)^34,35^. The value of $\zeta$ depends on the deposition process and the process parameters. Most noticeably, the thermal hysteretic insulation–metal–insulator transition is affected by polycrystallinity^74^. As our proposed designs avoid the temperature-regime of hysteresis, we expect that their performance is going to be affected only to a limited degree by $\vert \zeta -2\vert \le 0.1$. Therefore, with stochiometry engineering^8^, vanadium-based materials^74,75^ have a high potential in novel physical scenarios and related applications, which still need to be explored.

## Methods

An intuitive design approach, based on the qualitative analysis of the dominant physics and simple estimates, was applied. CST Studio Suite^62^, a commercial software was used for numerical simulations. It is based on the finite integration method with controllable convergence and accuracy, and is particularly appropriate for unitary metasurfaces comprising unit cells of complex geometry. The frequency-domain solver and unit-cell (Floquet–Bloch) boundary conditions were adopted along with a tetrahedral mesh for simulations.

## References

[1] Kamali SM, Arbabi E, Arbabi A, Faraon A (2018). A review of dielectric optical metasurfaces for wavefront control. Nanophotonics.

[2] Imada M, Fujimori A, Tokura Y (1998). Metal-insulator transitions. Rev. Mod. Phys..

[3] Mott NF (1968). Metal-insulator transition. Rev. Mod. Phys..

[4] Wang Q, Rogers ETF, Gholipour B, Wang C-M, Yuan G, Teng J, Zheludev NI (2016). Optically reconfigurable metasurfaces and photonic devices based on phase change materials. Nat. Photon..

[5] Vendik IB, Vendik OG, Odit MA, Kholodnyak DV, Zubko SP, Sitnikova MF, Turalchuk PA, Zemlyakov KN, Munina IV, Kozlov DS, Turgaliev VM, Ustinov AB, Park Y, Kihm J, Lee C-W (2012). Tunable metamaterials for controlling THz radiation. IEEE Trans. Terahertz Sci. Technol..

[6] Adler D (1968). Mechanisms for metal-nonmetal transitions in transition-metal oxides and sulfides. Rev. Mod. Phys..

[7] Hyland GJ (1968). Some remarks on electronic phase transitions and on the nature of the ‘metallic’ state in VO$$_2$$. Rev. Mod. Phys..

[8] Shi R, Chen Y, Cai X, Lian Q, Zhang Z, Shen N, Amini A, Wang N, Cheng C (2021). Phase management in single-crystalline vanadium dioxide beams. Nat. Commun..

[9] Kakiuchida H, Jin P, Nakao S, Tazawa M (2007). Optical properties of vanadium dioxide films during semiconductive-metallic phase transition. Jpn. J. Appl. Phys..

[10] Son TV, Zongo K, Ba C, Beydaghyan G, Haché A (2014). Pure optical phase control with vanadium dioxide thin films. Opt. Commun..

[11] Cormier P, Son TV, Thibodeau J, Doucet A, Truong V-V, Haché A (2017). Vanadium dioxide as a material to control light polarization in the visible and near infrared. Opt. Commun..

[12] Rensberg J, Zhang S, Zhou Y, McLeod AS, Schwarz C, Goldflam M, Liu M, Kerbusch J, Nawrodt R, Ramanathan S, Basov DN, Capasso F, Ronning C, Kats MA (2016). Active optical metasurfaces based on defect-engineered phase-transition materials. Nano Lett..

[13] Kim M, Jeong J, Poon JKS, Eleftheriades GV (2016). Vanadium-dioxide-assisted digital optical metasurfaces for dynamic wavefront engineering. J. Opt. Soc. Am. B.

[14] Nouman MT, Hwang JH, Faiyaz M, Lee K-J, Noh D-Y, Jang JH (2018). Vanadium dioxide based frequency tunable metasurface filters for realizing reconfigurable terahertz optical phase and polarization control. Opt. Express.

[15] Wang S, Cai C, You M, Liu F, Wu M, Li S, Bao H, Kang L, Werner DH (2019). Vanadium dioxide based broadband THz metamaterial absorbers with high tunability: Simulation study. Opt. Express.

[16] Momoda LA (2004). The future of engineering materials: Multifunction for performance-tailored structures. Bridge.

[17] Lakhtakia A (2015). From bioinspired multifunctionality to mimumes. Bioinsp. Biomim. Nanobiomater..

[18] Kumar P, Rai S, Bhattacharyya S, Lakhtakia A, Jain PK (2020). Graphene-sandwich metasurface as a frequency shifter, switch, and isolator at terahertz frequencies. Opt. Eng..

[19] Shen N, Chen S, Huang R, Huang J, Li J, Shi R, Niu S, Amini A, Cheng C (2021). Vanadium dioxide for thermochromic smart windows in ambient conditions. Mater. Today Energy.

[20] Shi R, Shen N, Wang J, Wang W, Amini A, Wang N, Cheng C (2019). Recent advances in fabrication strategies, phase transition modulation, and advanced applications of vanadium dioxide. Appl. Phys. Rev..

[21] Luo H, Wang B, Wang E, Wang X, Sun Y, Li Q, Fan S, Cheng C, Lu K (2019). Phase-transition modulated, high-performance dual-mode photodetectors based on WSe$$_2$$/VO$$_2$$ heterojunctions. Appl. Phys. Rev..

[22] Zheng X, Xiao Z, Ling X (2018). A tunable hybrid metamaterial reflective polarization converter based on vanadium oxide film. Plasmonics.

[23] Singh R, Azad AK, Jia QX, Taylor AJ, Chen H-T (2011). Thermal tunability in terahertz metamaterials fabricated on strontium titanate single-crystal substrates. Opt. Lett..

[24] Kats MA, Sharma D, Lin J, Genevet P, Blanchard R, Yang Z, Qazilbash MM, Basov DN, Ramanathan S, Capasso F (2012). Ultra-thin perfect absorber employing a tunable phase change material. Appl. Phys. Lett..

[25] Seo M, Kyoung J, Park H, Koo S, Kim H-S, Bernien H, Kim BJ, Choe JH, Ahn YH, Kim H-T, Park N, Park Q-H, Ahn K, Kim D-S (2010). Active terahertz nanoantennas based on $$\text{ VO}_2$$ phase transition. Nano Lett..

[26] Kocer H, Ozer A, Butun S, Wang K, Wu J, Kurt H, Aydin K (2019). Thermally tuning infrared light scattering using planar layered thin films and space gradient metasurface. IEEE J. Selec. Top. Quan. Electron..

[27] Lai W, Shi R, Yuan H, Liu G, Amini A, Cheng C (2021). Fully optically tunable and flexible composite films for enhanced terahertz control and multifunctional terahertz devices. ACS Appl. Electron. Mater..

[28] Han J, Lakhtakia A (2009). Semiconductor split-ring resonators for thermally tunable terahertz metamaterials. J. Mod. Opt..

[29] Lv TT, Li YX, Ma HF, Zhu Z, Li ZP, Guan CY, Shi JH, Zhang H, Cui TJ (2016). Hybrid metamaterial switching for manipulating chirality based on VO$$_2$$ phase transition. Sci. Rep..

[30] Wang D, Zhang L, Gong Y, Jian L, Venkatesan T, Qiu C-W, Hong M (2016). Multiband switchable terahertz quarter-wave plates via phase-change metasurfaces. IEEE Photon. J..

[31] Zhang C, Zhou G, Wu J, Tang Y, Wen Q, Li S, Han J, Jin B, Chen J, Wu P (2019). Active control of terahertz waves using vanadium-dioxide-embedded metamaterials. Phys. Rev. Appl..

[32] Liu M, Xu Q, Chen X, Plum E, Li H, Zhang X, Zhang C, Zou C, Han J, Zhang W (2019). Temperature-controlled asymmetric transmission of electromagnetic waves. Sci. Rep..

[33] Liu X, Wang Q, Zhang X, Li H, Xu Q, Xu Y, Chen X, Li S, Liu M, Tian Z, Zhang C, Zou C, Han J, Zhang W (2019). Thermally dependent dynamic meta-holography using a vanadium dioxide integrated metasurface. Adv. Opt. Mater..

[34] Motyka MA, Gauntt BD, Horn MW, Podraza NJ (2012). Microstructural evolution of thin film vanadium oxide prepared by pulsed-dc magnetron sputtering. J. Appl. Phys..

[35] Basantani HA, Kozlowski S, Lee M-Y, Li J, Bharadwaja SSN, Dickey EC, Jackson TN, Horn M (2012). Enhanced electrical and noise properties of nanocomposite vanadium oxide thin films by reactive pulsed-dc magnetron sputtering. Appl. Phys. Lett..

[36] Němec H, Kužel P, Kadlec F, Kadlec C, Yahiaoui R, Mounaix P (2009). Tunable terahertz metamaterials with negative permeability. Phys. Rev. B.

[37] Serebryannikov AE, Lakhtakia A, Ozbay E (2016). Single and cascaded, magnetically controllable metasurfaces as terahertz filters. J. Opt. Soc. Am. B.

[38] Kumar P, Lakhtakia A, Jain PK (2020). Tricontrollable pixelated metasurface for stopband for terahertz radiation. J. Electromag. Waves Appl..

[39] Xing Q, Wang C, Huang S, Liu T, Xie Y, Song C, Wang F, Li X, Zhou L, Yan H (2020). Tunable graphene split-ring resonators. Phys. Rev. Appl..

[40] Folland TG, Fali A, White ST, Matson JR, Liu S, Aghamiri NA, Edgar JH, Haglund RF, Abate Y, Caldwell JD (2018). Reconfigurable infrared hyperbolic metasurfaces using phase change materials. Nat. Commun..

[41] Wen Q-Y, Zhang H-W, Yang Q-H, Xie Y-S, Chen K, Liu Y-L (2010). Terahertz metamaterials with $$\text{ VO}_{2}$$ cut-wires for thermal tunability. Appl. Phys. Lett..

[42] Shelton DJ, Coffey KR, Boreman GD (2010). Experimental demonstration of tunable phase in a thermochromic infrared-reflectarray metamaterial. Opt. Express.

[43] Paik T, Hong S-H, Gaulding EA, Caglayan H, Gordon TR, Engheta N, Kagan CR, Murray CB (2014). Solution-processed phase-change $$\text{ VO}_{2}$$ metamaterials from colloidal vanadium oxide (VO$$_{\rm x}$$) nanocrystals. ACS Nano.

[44] Song Z, Zhang J (2020). Achieving broadband absorption and polarization conversion with a vanadium dioxide metasurface in the same terahertz frequencies. Opt. Express.

[45] Xu Z, Song Z (2021). $$\text{ VO}_{2}$$-based switchable metasurface with broadband photonic spin hall effect and absorption. IEEE Photon. J..

[46] Li X, Tang S, Ding F, Zhong S, Yang Y, Jiang T, Zhou J (2019). Switchable multifunctional terahertz metasurfaces employing vanadium dioxide. Sci. Rep..

[47] Mao M, Liang Y, Liang R, Zhao L, Xu N, Guo J, Wang F, Meng H, Liu H, Wei Z (2019). Dynamically temperature-voltage controlled multifunctional device based on $$\text{ VO}_{2}$$ and graphene hybrid metamaterials: Perfect absorber and highly efficient polarization converter. NanomatERIALS (Basel).

[48] Wang T, He J-W, Guo J, Wang X, Feng S, Kuhl F, Becker M, Polity A, Klar PJ, Zhang Y (2019). Thermally switchable terahertz wavefront metasurface modulators based on the insulator-to-metal transition of vanadium dioxide. Opt. Express.

[49] Zhang M, Song Z (2021). Switchable terahertz metamaterial absorber with broadband absorption and multiband absorption. Opt. Express.

[50] Dong K, Hong S, Deng Y, Ma H, Li J, Wang X, Yeo J, Wang L, Lou S, Tom KB, Liu K, You Z, Wei Y, Grigoropoulos CP, Yao J, Wu J (2018). A lithography-free and field-programmable photonic metacanvas. Adv. Mater..

[51] Zouros GP, Kolezas GD, Almpanis E, Baskourelos K, Stefański TP, Tsakmakidis KL (2020). Magnetic switching of Kerker scattering in spherical microresonators. Nanophoton..

[52] Lewi T, Evans HA, Butakov NA, Schuller JA (2017). Ultrawide thermo-optic tuning of PbTe meta-atoms. Nano Lett..

[53] Serebryannikov AE, Alici KB, Ozbay E, Lakhtakia A (2018). Thermally sensitive scattering of terahertz waves by coated cylinders for tunable invisibility and masking. Opt. Express.

[54] Lepeshov S, Krasnok A, Alù A (2019). Nonscattering-to-superscattering switch with phase-change materials. ACS Photon..

[55] Leitis A, Heßler A, Wahl S, Wuttig M, Taubner T, Tittl A, Altug H (2020). All-dielectric programmable huygens’ metasurfaces. Adv. Funct. Mater..

[56] Vorontsov AM, Paramonov PV, Valley MT, Vorontsov MA (2008). Generation of infinitely long phase screens for modeling of optical wave propagation in atmospheric turbulence. Waves Random Complex Media.

[57] Menzel C, Helgert C, Rockstuhl C, Kley E-B, Tunnermann A, Pertsch T, Lederer F (2010). Asymmetric transmission of linearly polarized light at optical metamaterials. Phys. Rev. Lett..

[58] Cheng Y, Nie Y, Wang X, Gong R (2013). An ultrathin transparent metamaterial polarization transformer based on a twist-split-ring resonator. Appl. Phys. A.

[59] Mutlu M, Akosman AE, Serebryannikov AE, Ozbay E (2012). Diodelike asymmetric transmission of linearly polarized waves using magnetoelectric coupling and electromagnetic wave tunneling. Phys. Rev. Lett..

[60] Serebryannikov AE, Lakhtakia A, Aalizadeh M, Ozbay E, Vandenbosch GAE (2018). Temperature-mediated invocation of the vacuum state for switchable ultrawide-angle and broadband deflection. Sci. Rep..

[61] Grady NK, Heyes JE, Roy Chowdhury D, Zeng Y, Reiten MT, Azad AK, Taylor AJ, Dalvit DAR, Chen H-T (2013). Terahertz metamaterials for linear polarization conversion and anomalous refraction. Science.

[62] CST Studio Suite. https://www.3ds.com/products-services/simulia/products/cst-studio-suite/solvers/.

[63] Mackay TG, Lakhtakia A (2019). Electromagnetic Anisotropy and Bianisotropy.

[64] Wang D, Zhang L, Gu Y, Mehmood MQ, Gong Y, Srivastava A, Jian L, Venkatesan T, Qiu C-W, Hong M (2015). Switchable ultrathin quarter-wave plate in terahertz using active phase-change metasurface. Sci. Rep..

[65] Wang Q, Gao B, Raglione M, Wang H, Li B, Toor F, Arnold MA, Ding F (2019). Design, fabrication, and modulation of THz bandpass metamaterials. Laser Photon. Rev..

[66] Liu W, Chen S, Li Z, Cheng H, Yu P, Li J, Tian J (2015). Realization of broadband cross-polarization conversion in transmission mode in the terahertz region using a single-layer metasurface. Opt. Lett..

[67] Zhang K, Yuan Y, Ding X, Li H, Ratni B, Wu Q, Liu J, Burokur SN, Tan J (2021). Polarization-engineered noninterleaved metasurface for integer and fractional orbital angular momentum multiplexing. Laser Photon. Rev..

[68] Yuan Y, Zhang K, Ratni B, Song Q, Ding X, Wu Q, Burokur SN, Genevet P (2020). Independent phase modulation for quadruplex polarization channels enabled by chirality-assisted geometric-phase metasurfaces. Nat. Commun..

[69] Chang C-C, Headland D, Abbott D, Withayachumnankul W, Chen H-T (2017). Demonstration of a highly efficient terahertz flat lens employing tri-layer metasurfaces. Opt. Lett..

[70] Rodríguez-Ulibarri P, Beruete M, Navarro-Cía M, Serebryannikov AE (2013). Wideband unidirectional transmission with tunable sign-switchable refraction and deflection in nonsymmetric structures. Phys. Rev. B.

[71] Wong JPS, Epstein A, Eleftheriades GV (2016). Reflectionless wide-angle refracting metasurfaces. IEEE Antennas Wirel. Propagat. Lett..

[72] Cheng Y, Fan J, Luo H, Chen F, Feng N, Mao X, Gong R (2019). Dual-band and high-efficiency circular polarization conversion via asymmetric transmission with anisotropic metamaterial in the terahertz region. Opt. Mater. Express.

[73] Liu D-J, Xiao Z-Y, Ma X-L, Wang Z-H (2015). Broadband asymmetric transmission and multi-band $$90^{\circ }$$ polarization rotator of linearly polarized wave based on multi-layered metamaterial. Opt. Commun..

[74] Subramanyam G, Shin E, Peri PR, Katiyar R, Naziripour G, Dey S, Subramanyam G, Banerjee PP, Gudmundsson KS, Lakhtakia A (2021). Structural, electrical, and electromagnetic properties of nanostructured vanadium dioxide thin films. Thin Film Nanophoton..

[75] Wei Q, DeBlock RH, Butts DM, Choi C, Dunn B (2020). Pseudocapacitive vanadium-based materials toward high-rate sodium-ion storage. Energy Environ. Mat..

